# Fel d 1 surface expression on plant‐made *e*Bioparticles combines potent immune activation and hypoallergenicity

**DOI:** 10.1111/all.15464

**Published:** 2022-08-08

**Authors:** Stefanie Busold, Lorenz Aglas, Charlotte Menage, Lydia Auger, Réjean Desgagnés, Loïc Faye, Anne‐Catherine Fitchette, Esther C. de Jong, Caroline Martel, Maria Stigler, Virginie Catala‐Stordeur, Guy Tropper, Louis‐Philippe Vézina, Véronique Gomord, Teunis B. H. Geijtenbeek, Ronald van Ree

**Affiliations:** ^1^ Department of Experimental Immunology Amsterdam UMC Location University of Amsterdam Amsterdam The Netherlands; ^2^ Amsterdam Institute for Infection and Immunity,Inflammatory Diseases Amsterdam The Netherlands; ^3^ Department of Biosciences and Medical Biology Paris Lodron University of Salzburg Salzburg Austria; ^4^ Angany Inc. Québec Québec Canada; ^5^ Angany Innovation Val‐de‐Reuil France; ^6^ Department of Otorhinolaryngology Amsterdam UMC location University of Amsterdam Amsterdam The Netherlands

## AUTHOR CONTRIBUTIONS

S. Busold, L. Aglas, C. Menage, and M. Stigler contributed to this study by designing the experiments and acquiring, interpreting, and analyzing the data. L. Auger, R. Desgagnés, L. Faye, A.‐C. Fitchette, C. Martel, V. Catala‐Stordeur, G. Tropper, L.‐P. Vézina, and V. Gomord contributed to the concept design, development, or manufacturing of the plant‐derived bioparticles platform. S. Busold, L. Aglas, V. Gomord, T. B. H. Geijtenbeek, and R. van Ree wrote, revised, and edited the manuscript. E. C. de Jong, T. B. H. Geijtenbeek, and R. van Ree supervised the study. All authors critically reviewed the manuscript.

## FUNDING INFORMATION

This project is embedded in the DC4Balance consortium, which was supported by Health Holland. The work of the authors has further been supported by Angany Inc. (Quebec, Canada), the Austrian Science Funds (Projects P32189), and the University of Salzburg priority program “Allergy‐Cancer‐BioNano Research Centre.”

## CONFLICT OF INTEREST

Stefanie Busold and Charlotte Menage received contract research funding and study material from Angany Inc. and the DC4Balance consortium. Lorenz Aglas and Maria Stigler received contract research funding and study material from Angany Inc. Louis‐P Vézina is a cofounder, board member, and CEO of Angany Inc. Lydia Auger, Réjean Desgagnés, Loïc Faye, Anne‐Catherine Fitchette, Caroline Martel, Virginie Stordeur, Guy Tropper, and Véronique Gomord are employees of Angany Inc. Esther C. de Jong and Teunis B. H. Geijtenbeek received funding from the DC4Balance consortium. Ronald van Ree received contract research funding and research material from Angany Inc. and the DC4Balance consortium and besides received consulting fees and/or speaker's fees from Angany Inc., HAL Allergy BV, and Citeq BV, ThermoFisher Scientific, Reacta Healthcare Ltd., Mission MightyMe, and AB Enzymes and has stock options from Angany Inc.

To the Editor,

Allergen immunotherapy (AIT) still remains the only disease‐modifying treatment for immunoglobulin E (IgE)‐mediated allergies with proven sustained efficacy upon discontinuation of the treatment, provided it is administered regularly for at least 3 years. Aluminum salts still remain a common depot and adjuvant for subcutaneous AIT that moderately reduce allergenicity; however, the risk of severe allergic reactions persists, making careful updosing protocols necessary.^1^ These characteristics justify the need for shorter treatments with improved safety profiles.

**FIGURE 1:**
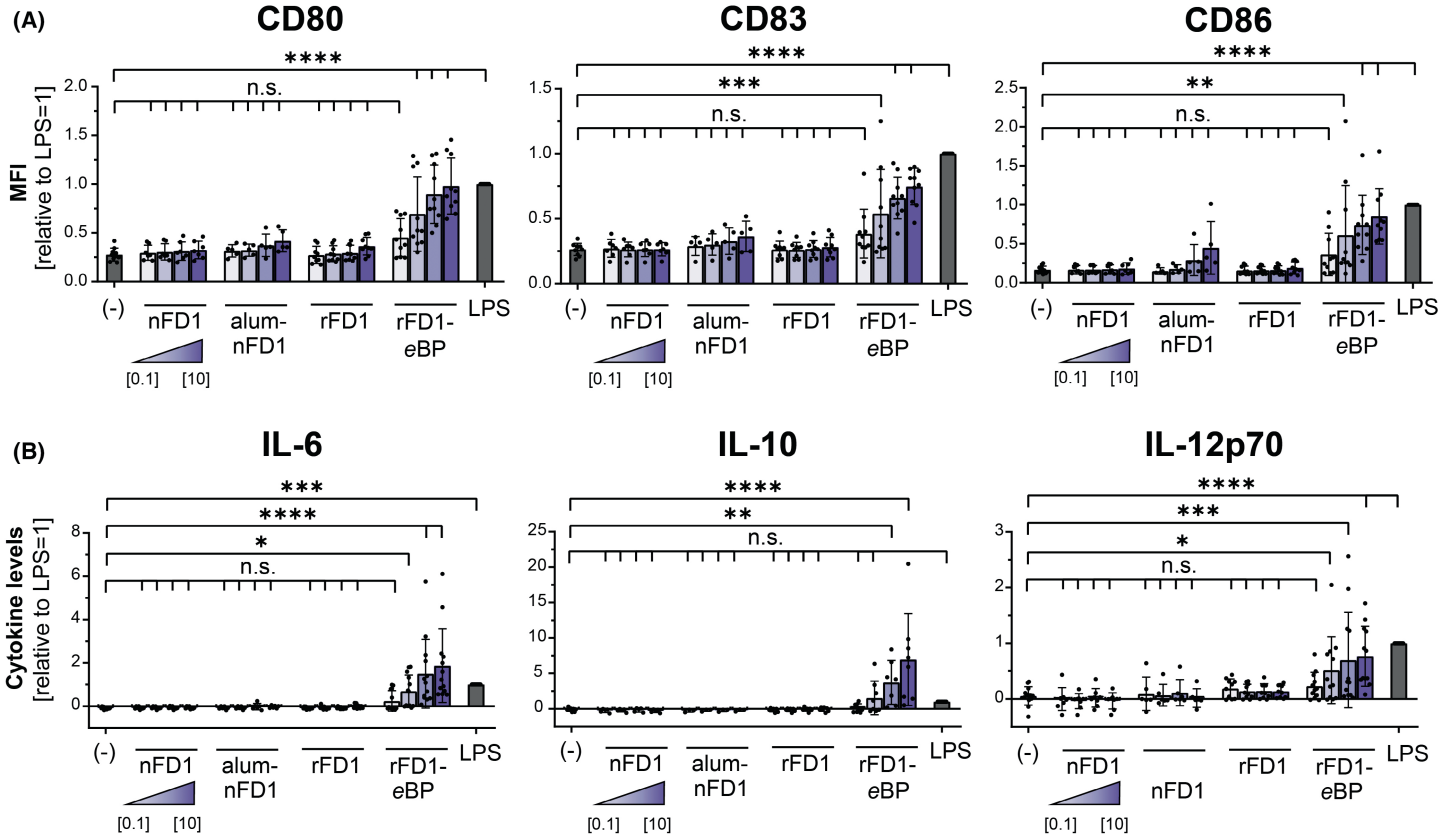
Enveloped bioparticles surface‐displaying Fel d 1 induce moDC maturation. MoDCs were stimulated for 24 h with either natural soluble, natural alum‐adsorbed, recombinant soluble, or recombinant Fel d 1 displayed on *e*BPs (nFD1/alum‐nFD1/ rFD1/rFD1‐*e*BP) at concentrations of 0.1, 1, 3, and 10 μg/ml, respectively. (A) MoDC activation markers were measured using flow cytometry. The measured mean fluorescent intensity (MFI) data are shown relative to levels obtained from cells stimulated for 24 h with 10 ng/ml lipopolysaccharide (LPS). The data are presented as the mean ± SD (*n* = 5–10 donors). B) Culture supernatants were harvested, and the levels of secreted IL‐6, IL‐10, and IL‐12p70 were measured by ELISA. The measured data are shown relative to levels obtained from cells stimulated for 24 h with 10 ng/ml lipopolysaccharide (LPS). The data are presented as the mean ± SD (*n* = 3–13 donors). n.s. *p* > .05, * *p* < .05, ** *p* < .01, *** *p* < .001, **** *p* < .0001 relative to unstimulated cells (one‐way ANOVA with Dunnett's post hoc analysis test).

Here, we describe plant‐made enveloped *e*Bioparticles (*e*BPs) surface‐displaying high‐density recombinant allergen as a novel AIT platform (Appendix S1). Previous in vivo experiments indicate that this *e*BP platform induces a robust protective IgG response more potently than the alum‐adsorbed allergen.^2^ In this study, we assessed immunogenicity and allergenicity of *e*BPs displaying the major cat allergen Fel d 1 in a human in vitro setting. As dendritic cells are the most potent inducers and orchestrators of immune responses, we treated human monocyte‐derived dendritic cells (moDCs) with equivalent concentrations of soluble natural and recombinant, alum‐adsorbed natural, and *e*BP‐displayed recombinant Fel d 1. In contrast to soluble and alum‐adsorbed Fel d 1, *e*BP presentation significantly upregulated the DC surface activation markers CD80, CD83, and CD86 already at low concentrations (1 μg/ml, Figure 1A). In line with these results, only stimulation with Fel d 1‐displaying *e*BPs induced a potent production of interleukin (IL)‐6, IL‐10, and IL‐12p70 (Figure 1B). Our data therefore strongly suggest that plant‐derived *e*BPs are efficient immune activators. Immune‐stimulatory properties are required to reprogram the allergic T‐helper‐cell type 2 (Th2) response. The observed IL‐10 and IL‐12 responses may hence contribute to the rapid establishment of an allergen‐specific mixed Th1/Treg response. Even though the mechanism of the immune‐stimulatory properties remains speculative, it appears likely that high‐density allergen presentation results in better crosslinking of DC surface receptors accompanied by preferential uptake due to the particulate presentation.

**FIGURE 2:**
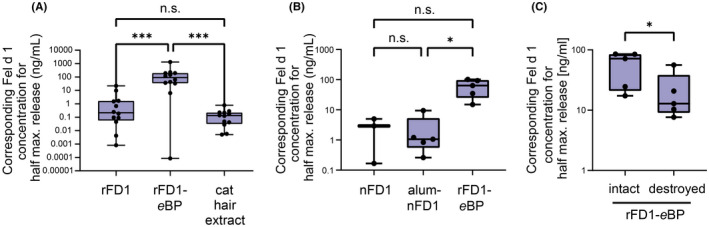
Enveloped bioparticles surface‐displaying Fel d 1 are hypoallergenic. FcεRI‐expressing RBL‐2H3 cells were sensitized with complement‐inactivated sera from cat‐allergic patients and subsequently stimulated for 1 h at 37°C with (A) soluble recombinant Fel d 1 (rFD1), enveloped bioparticles displaying recombinant Fel d 1 (rFD1‐*e*BP), or cat hair extract; (B) natural soluble, natural alum‐adsorbed, or recombinant bioparticles‐associated Fel d 1 (nFD1/alum‐nFD1/rFD1‐*e*BP); or (C) intact compared with destroyed Fel d 1‐displaying bioparticles (rFD1‐*e*BP). As a readout for basophil degranulation, the collected supernatants were screened for β‐hexosaminidase activity by incubation with the fluorogenic substrate 4‐methyl umbelliferyl‐N‐acetyl‐beta‐D‐glucosaminide. The data were corrected for spontaneous release (unstimulated cells) and normalized to the maximal enzyme release (100%) caused by cell lysis using 10% Triton X‐100. The data are presented as the median ± SD. (A) *n* = 12 sera (repeated‐measures one‐way ANOVA with Tukey's post hoc analysis test), (B) *n* = 3–5 sera (mixed‐effects analysis with Tukey's post hoc analysis test), (C) n = 5 sera (paired *t*‐test). n.s. *p* > .05, *p < .05, ** *p* < .01, *** *p* < .001, **** *p* < .0001.

Furthermore, we assessed the allergenicity of the *e*BPs by measuring β‐hexosaminidase secretion of rat basophil leukemia (RBL‐2H3) cells presensitized with human IgE from Fel d 1‐sensitized patients. Soluble plant‐produced recombinant Fel d 1 induced basophil degranulation in a comparable concentration range as observed for Fel d 1 in cat dander extract, demonstrating that the recombinant allergen is a good mimic of the native protein (Figure 2A). By contrast, >400‐fold and 60‐fold higher median Fel d 1 concentration on the *e*BPs were needed to trigger half maximum β‐hexosaminidase release, compared with soluble recombinant Fel d 1 and alum‐adsorbed natural Fel d 1, respectively (Figure 2B). To establish the importance of *e*BP structure for hypoallergenicity, we purposely maltreated the *e*BPs by a combination of vortexing and sonication. The treatment resulted in >5‐fold increased allergenicity compared with intact *e*BPs, highlighting a significant involvement of the particulate 3D ultrastructure in the hypoallergenic properties (Figure 2C). These data demonstrate that multivalent high‐density presentation of Fel d 1 on *e*BPs potently reduces allergenicity, not only compared with soluble but also with alum‐adsorbed Fel d 1. Earlier publications based on virus‐like particles displaying allergens have reported similar hypoallergenic outcomes for 3D allergen presentation.^3^, ^4^ Physical and biochemical aspects of repetitive surface distribution and resulting changes in allergen accessibility might serve as a possible explanation for the observed hypoallergenic outcome. While repetitively displayed allergens appear to have an inhibitory effect on basophils and mast cells, B‐cell responses can still be triggered.^3^, ^5^ Based on the current and previous findings, plant‐derived *e*BPs are promising AIT candidates showing enhanced immunogenicity in the absence of anaphylaxis. The endogenous adjuvant properties of in vivo‐assembled plant *e*BPs may harbor the potential to make updosing protocols obsolete and instead facilitate high‐dose prime‐boost approaches.

## Supporting information


Appendix S1

## Data Availability

Research data are not shared.
