# Supplementary material for: Fel d 1 surface expression on plant‐made eBioparticles combines potent immune activation and hypoallergenicity
Source: Allergy. 2022 Aug 8;77(10):3124–6. doi: 10.1111/all.15464 (PMC10286754; doi:10.1111/all.15464)
Supplement: Supplementary file 1 — Appendix S1 [file ALL-77-3124-s001.pdf]

Supplementary information

S.1_Supporting Methods

Allergens and bioparticles

Purified natural Fel d 1 was acquired from Inbio (Charlottesville, VA, USA). Alum adsorption was done by pre-incubating the natural Fel d 1 (0.8 mg/mL) for 45 minutes with Imject^{TM} Alum Adjuvant (40 mg/mL aluminum hydroxide and 40 mg/mL magnesium hydroxide; Thermo Fisher, Rockford, USA) at an alum:allergen volume ratio of 1:4.

Purified recombinant soluble and *e*BP-associated Fel d 1 were produced by recombinant transient gene expression in plant cells (Angany Innovation, Val de Reuil, France) as described in detail by V. Gomord and colleagues[1], (V. Gomord in preparation). In short, for the expression of soluble recombinant Fel d 1 the complementary DNA (cDNA) encoding the tobacco chitinase signal sequence (accession number: QEQ12695) was fused at the N-terminal end of the Fel d 1 chain 1 (accession number: AAC37318.1) and at the N-terminal end of the Fel d 1 chain 2 (accession number : NP_001041619.1), respectively. A cDNA sequence coding for hexa-histidine/FLAG TAG was added at the C-terminus. In contrast, *e*BPs displaying recombinant Fel d 1 were synthesized by introducing a fusion construct containing chain 1 and chain 2 of Fel d 1 (V. Gomord in preparation), framed by the tobacco chitinase signal sequence at the N-terminus and the coiled-coil yeast transcription factor GNC4 (accession number: 1GCM_A) fused to the TM/CT domain of influenza virus hemagglutinin H5N1 (accession number: ABW06108.1) at the C-terminus. The respective vectors were then introduced into *Agrobacterium tumefaciens* strain LBA4404, which was subsequently used to transfect *Nicotiana benthamiana* for transient gene expression. Following protein expression and modification in the plant cell secretory pathway, the encoded membrane protein carrier sequence induces budding of allergen-covered *e*BPs from the plant cell's plasma membrane, which are harvested and purified by a combination of tangential flow filtration and size exclusion chromatography (Figure S.2). The results are fully *in vivo* synthesized *e*BPs of an approximate size of around 150 nm.

Generation and stimulation of dendritic cells

To generate suspension-growing moDCs, we isolated monocytes from buffy coats of blood bank donors (Sanquin Bloodbank, Amsterdam, The Netherlands) as described previously[2]. The isolated monocytes were cultured for six days at 37 °C and 5% CO_2 atmosphere in RPMI 1640 (Gibco/Life Technologies, New York, USA) supplemented with 10% fetal calf serum, L-glutamine (2 mM, Lonza, Verviers, Belgium), pen/strep (100 U/mL and 0.1 mg/mL, respectively; Gibco/Life Technologies), interleukin 4 (IL-4, 25 pg/mL, Gibco) and GM-CSF (20 pg/mL, Gibco/Life Technologies), driving the differentiation into moDCs. Immature moDCs were then treated with medium (-), natural Fel d 1, alum-adsorbed natural Fel d 1, recombinant Fel d 1, or *e*BPs displaying recombinant Fel d 1 at concentrations of 0.1-10 µg/mL or, as positive control with 10 ng/mL lipopolysaccharides (LPS) derived from *Salmonella typhosa* (Sigma-Aldrich, St. Louis, MO, USA). The allergen concentration of the *e*BPs was adjusted to the concentration of soluble allergen by Western blot quantification as described before[1].

Flow cytometry analysis

To assess moDC activation and viability, we harvested moDCs 24 h post-stimulation. To quantify surface expression of DC maturation markers, we stained for 30 min at 4 °C with PE-conjugated mouse anti-CD80, APC-conjugated mouse anti-CD83 and FITC-conjugated mouse anti-CD86 (all BD Biosciences, New Jersey, USA) diluted in phosphate-buffered saline (PBS) supplemented with 0.5% bovine serum albumin and 0.02% sodium azide.

We analysed cellular viability using the Annexin V apoptosis detection Kit (Invitrogen, Vienna, Austria) according to the manufacturer's instructions. In short, cells were incubated with Annexin V-FITC and counterstained using propidium iodide (PI), allowing for differentiation of viable cells from early apoptotic and necrotic cells (Figure S.3). As a positive control (+) we used moDCs that were incubated for 20 min at 56 °C.

Flow cytometry analysis was performed using the FACS Canto II (BD Biosciences) and FlowJo software v10. MoDC maturation data are shown as mean fluorescence intensity (MFI) relative to data obtained from LPS treatment (LPS=1). DC viability data represent percentages of cells within the respective group.

Cytokine secretion

We harvested supernatants from moDC cultures after 24 h of sample exposure and measured the secretion of IL-6, IL-10 and IL-12 p70 (Invitrogen) by ELISA. Cytokine secretion data are shown relative to data obtained from LPS treatment (LPS=1).

Basophil mediator release assay

We performed a mediator release assay measuring β-hexosaminidase release as readout for basophil degranulation to analyse allergenicity of allergen-displaying *e*BPs compared to the respective soluble allergen and allergenic extract. To that end, a rat basophil (RBL-2H3) cell line transfected with the human high-affinity IgE receptor (FcεRI) was used. RBL-2H3 cells (1 x 10^5 per well) were passively sensitized with human serum derived from cat allergic patients. Allergen-specific IgE concentrations for each serum are provided in table S.4. For complement inactivation, we pre-incubated sera with P3X63Ag8.653 cells (ATCC CRL-1580™, Manassas, VA, USA) prior to the overnight sensitization. Degranulation and associated β-hexosaminidase release were triggered by stimulating the RBL-2H3 cells either with (A) soluble recombinant Fel d 1, *e*BPs displaying recombinant Fel d 1, or cat dander extract, (B) soluble natural Fel d 1, alum-adsorbed natural Fel d 1, or *e*BPs displaying recombinant Fel d 1, or (C) intact and destructed *e*BPs displaying recombinant Fel d 1 for one hour at 37 °C, 7% CO_2, with antigen concentrations ranging from 3.4 fg/mL to 10 µg/mL. The antigen concentration of *e*BPs as well as of the cat dander extract was adjusted to the respective wild type allergen concentration by Western Blot using polyclonal sera of rabbits that were previously sensitized with Fel d 1. We detected β-hexosaminidase activity by incubation of supernatants from stimulated cells with the fluorogenic substrate 4-methyl umbelliferyl-N-acetyl-beta-D-glucosaminide (Sigma-Aldrich) and measured the fluorescence at an excitation and emission wavelength of 360 nm and 465 nm, respectively. Data were corrected for spontaneous release (unstimulated cells) and normalized to the maximal enzyme release (100%) caused by cell lysis using 10% Triton X-100 (Sigma-Aldrich). A "no antigen" control, consisting of serum-sensitized cells, which were not stimulated with an antigen, was included as baseline. To determine potential interferences caused by the storage buffer of the samples, antigen dilution series without cells were performed and incubated with the fluorogenic substrate. The two highest antigen concentrations of cat hair extract (10 and 0.5 µg/mL) resulted in a high background signal

(false positive), therefore, these concentrations were excluded from further analysis. The antigen concentration necessary to obtain half-maximal release was calculated by inserting the half-maximum release value into a logarithmic regression trend line. The half-maximum release was defined as half of the sum of the average of the maximal release values of each curve and the “no antigen” control [half-max. release = (average of max. release + “no antigen” control) / 2].

To exclude that observed differences in allergenicity are caused by interference of serum or allergen formulations, we assessed RBL cell viability. Our data show virtually no difference between pre-sensitized RBL-2H3 with and without further stimuli, while all conditions show highly significant differences to lysed cells (Fig. S.5).

Statistical analysis

For statistical analysis, GraphPad Prism version 8 (GraphPad Software LLC, San Diego, CA, USA) was used. To compare moDC reactivity relative to unstimulated baseline values, ordinary one-way ANOVA followed by Dunnett’s post-hoc analysis test was performed. For cell line-based mediator release assays, repeated-measures one-way ANOVA, mixed-effects analysis followed by Tukey’s post-hoc analysis test, or paired t-test was performed on transformed data (Y=log(Y)).

$P \geq 0.05$ was considered not significant (n.s.), * $P < 0.05$, ** $P < 0.01$, *** $P < 0.001$, and **** $P < 0.0001$).

References

1. *Gomord V et al. Design, production and immunomodulatory potency of a novel allergen bioparticle. PLoS One, 2020;15(12).*

2. *Mesman AW et al. Measles virus suppresses RIG-I-like receptor activation in dendritic cells via DC-SIGN-mediated inhibition of PP1 phosphatases. Cell Host Microbe. 2014;16(1):31-42.*

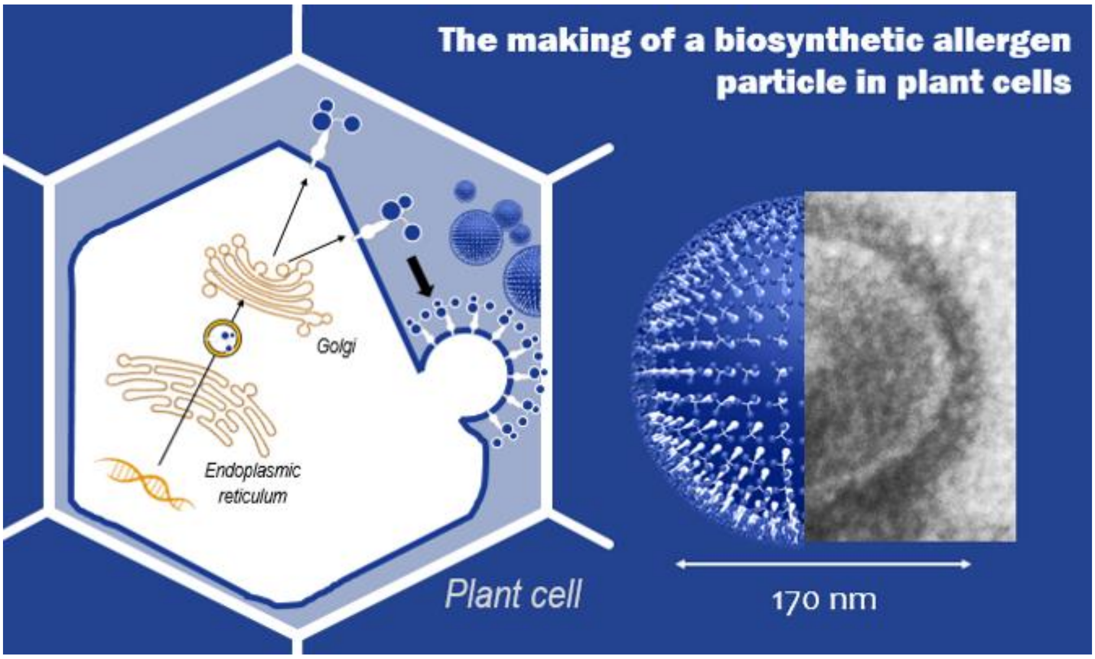

Figure S.2. Schematic overview of the production of plant-derived enveloped bioparticles.

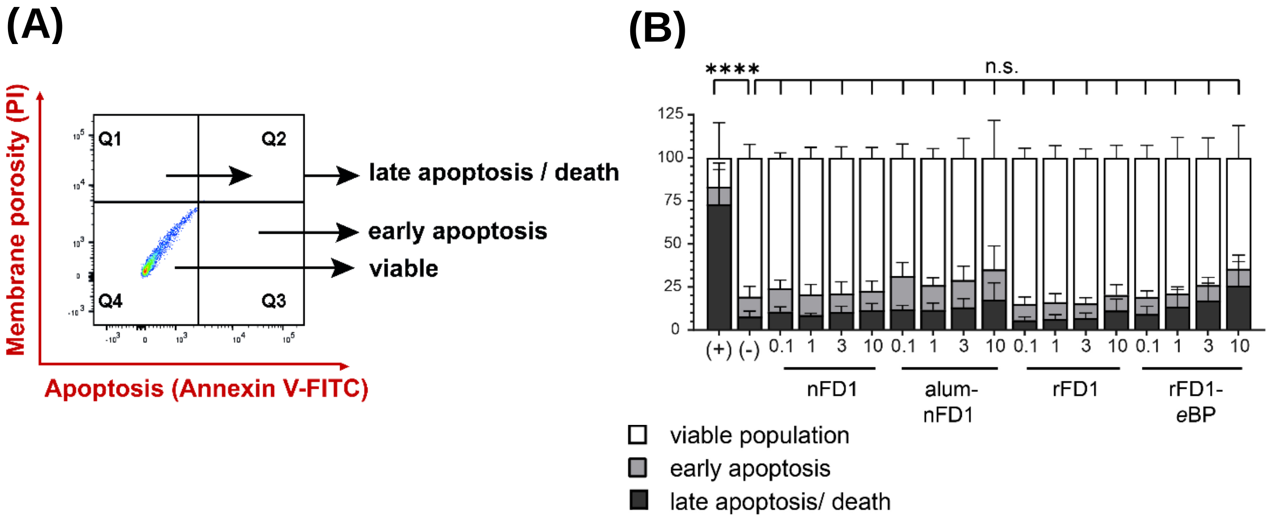

Figure S.3. **MoDC viability.** After 24 h of stimulation the cells were harvested and cell viability was assessed by flow cytometry. **(A)** Gating strategy: Based on the cellular location of phosphatidylserine, a binding partner for Annexins, the assay allows to distinguish between viable (Q4), early apoptotic (Q3), and late apoptotic/necrotic (Q1+Q2) cell populations. The plots were gates based on unstained controls. **(B)** Outcome viability assay after moDC stimulation with natural Fel d 1 (nFD1), alum-associated natural Fel d 1 (alum-nFD1), plant-made recombinant Feld 1 (rFD1) and *e*BPs displaying recombinant Fel d1 (rFD1-*e*BP). The data are presented as mean ± SD (n= 4-8 donors).
n.s. $p > 0.05$, * $p < 0.05$, ** $p < 0.01$, *** $p < 0.001$, **** $p < 0.0001$ relative to unstimulated cells (one-way ANOVA with Dunnett's post-hoc analysis test).

Table S.4. **Fel d 1-specific IgE levels in human test sera**

Figure	Serum	Serum IgE (kU/L)
2.a)	#1	32.74
	#2	83.46
	#3	18.39
	#4	17.63
	#5	16.73
	#6	31.58
	#7	50.37
	#8	17.6
	#9	34.32
	#10	18.94
	#11	26.06
	#12	17.26
2.b)	#1	32.74
	#4	17.63
	#13	61.96
	#14	19.52
	#15	21.09
2.c)	#1	32.74
	#4	17.63
	#13	61.96
	#16	16.93
	#15	21.09

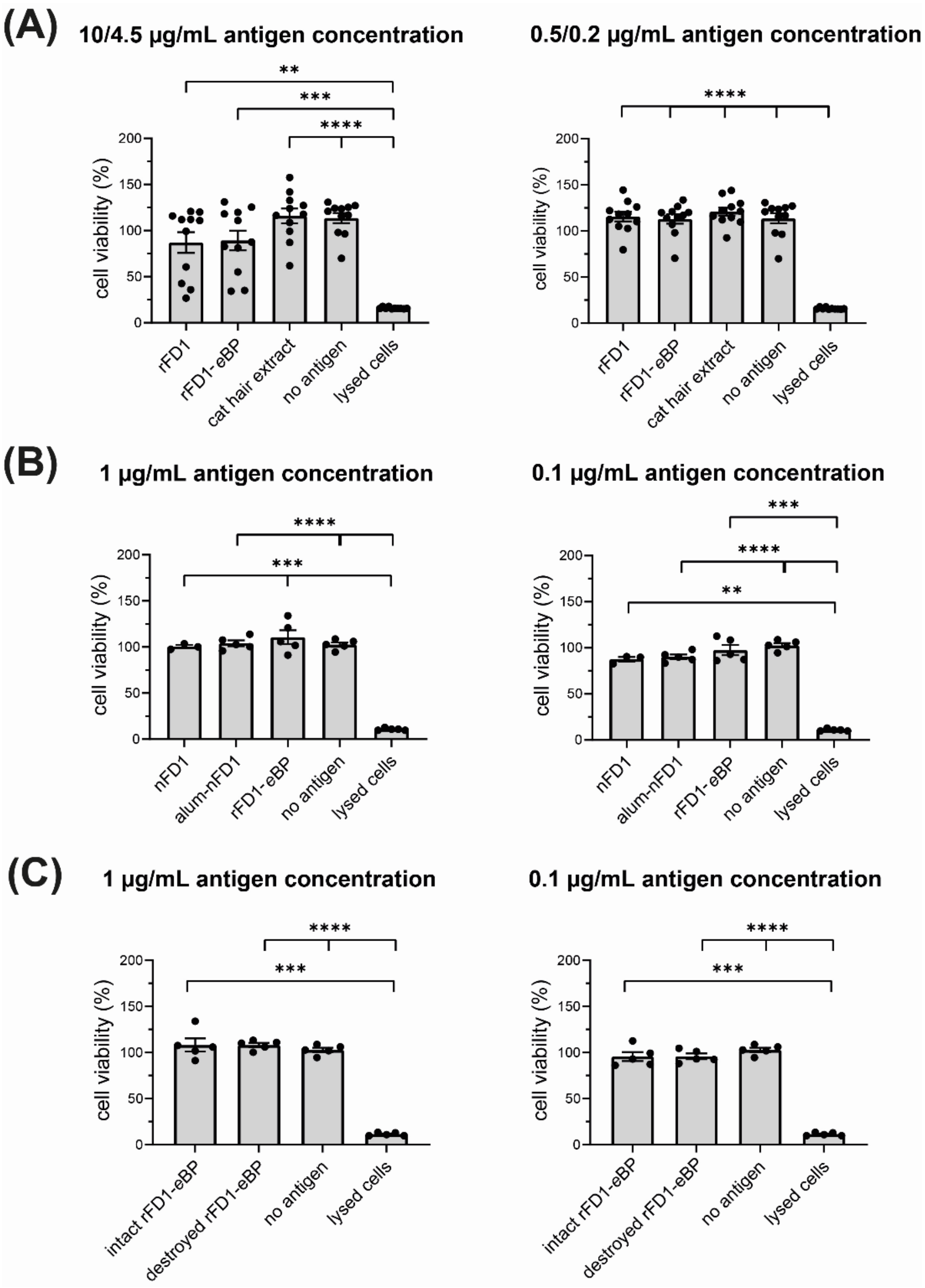

Figure S.5. **MTT viability assay on RBL-2H3.** MTT (3-(4,5-Dimethylthiazol-2-yl)-2,5-Diphenyltetrazolium Bromide, Sigma-Aldrich, Inc., St. Louis, MO, USA) assay was used to determine interference induced by cytotoxic effects of either the serum used for sensitization or the antigens used for stimulation: **(A)** soluble recombinant Fel d 1 (rFD1) and cat hair extract in the concentrations 10 µg/mL and 0.5 µg/mL; *e*BPs displaying recombinant Fel d 1 (rFD1-*e*BP) in the concentrations 4.5 µg/mL and 0.2 µg/mL **(B)** soluble natural Fel d 1 (nFD1), alum-adsorbed natural Fel d (alum-nFD1), and *e*BPs displaying recombinant Fel d 1 (rFD1-*e*BP) in the concentrations 1 µg/mL and 0.1 µg/mL.; **(C)** intact and destroyed *e*BPs displaying recombinant Fel d 1 (rFD1-*e*BP) in the concentrations 1 µg/mL and 0.1 µg/mL.

The data are presented as the mean ± SD (n=3-11 per group).

* $p < 0.05$, ** $p < 0.01$, *** $p < 0.001$, **** $p < 0.0001$ (Mixed-effects analysis/repeated measures one-way ANOVA)
